# Editorial: Nutrition, diet and allergic diseases

**DOI:** 10.3389/fnut.2023.1359005

**Published:** 2024-01-11

**Authors:** Sara Manti, Francesca Galletta, Salvatore Leonardi

**Affiliations:** ^1^Pediatric Unit, Department of Human Pathology of Adult and Childhood Gaetano Barresi, University of Messina, Messina, Italy; ^2^Pediatric Respiratory Unit, Department of Clinical and Experimental Medicine, University of Catania, Catania, Italy

**Keywords:** children, adults, allergic diseases, clinical outcomes, nutrition, immune system, diagnosis

The first 1,000 days of life are crucial in the growth and development of infants, as they have a diet of limited variability, mainly consisting of breastmilk and/or infant formula, followed by the introduction of milk and solid foods. The composition of all these foods significantly affects immune response and development, also affecting immunity later in life. Essentially, all dietary antigens are proteins that may prevent or contribute to the development of allergies.

*In vitro* and *in vivo* studies, especially in nutrition, increasingly require attention since they allow the identification and investigation of different and new treatments for allergic disorders. Accordingly, the notion that the composition and metabolic activity of foods and the role of intestinal microbiota in the development of allergies have become clearer over the last few years, and there is an urgent need to provide information on this issue.

The potential effects of vitamin D supplementation on airway obstruction may be through inflammatory cytokine changes, which are evident in T2-low asthma. In asthma, Zhou et al. first reported both *in vivo* and human models, outlining the associations between vitamin D levels and airway inflammation, airway resistance, and small-airway function (1). Similarly, supplementation with pomegranate, thanks to its anti-inflammatory properties, was significantly associated with an improvement in forced expiratory volume in 1 s (FEV1), FEV1/forced vital capacity (FVC) ratio (FEV1/FVC), and forced expiratory flow of 25–75% (FEF25–75%) in patients with mild and moderate allergic asthma (Shateri et al.). On the contrary, an increase in markers reflecting a pro-inflammatory immune response, such as High-density lipoprotein-cholesterol (HDL), folate, iron, and eosinophil levels, were independently and inversely associated with the immunological status of asthmatic adults (Wen, Zhuang et al.; Wen, Wang, Giri et al.; Wen, Wang, Xia et al.).

Globally, we urgently require an overview of best practices for biospecimen collection and analyses, as well as studies on the fundamentals of clinical data management (preparation and study startup including data collection, entry, cleaning, and authentication), and databases focusing on nutritional issues.

## Author contributions

SM: Conceptualization, Supervision, Validation, Visualization, Writing—original draft. FG: Visualization, Writing—review & editing. SL: Conceptualization, Supervision, Validation, Visualization, Writing—review & editing.
